# Age related clinical presentation and laboratory parameters in juvenile SLE: a Hungarian multicenter study

**DOI:** 10.1186/1546-0096-9-S1-P265

**Published:** 2011-09-14

**Authors:** B Derfalvi, A Malik, M Kreko, K Pasti, T Tarr, G Marton, Zs Gyorke, B Mosdosi, Z Nyul, J Noll, I Csurke, F Harangi, Zs Balogh, I Orban, K Sevcic, E Kiss, R Kaposzta, B Szucs, S Turi, P Sallay, Gy Reusz, T Tulassay, AJ Szabo

**Affiliations:** 1 2nd. Dept of Pediatrics, Semmelweis University Budapest; 2 1st. Dept. of Pediatrics, Semmelweis University Budapest; 3 3rd Department of Internal Medicine, University of Debrecen; 4 BAZ County Teaching Hospital, Miskolc; 5 Dept. of Pediatrics, POTE University of Medicine, Pecs; 6 Heim Pal Childrens’Hospital, Budapest; 7 Josa Andras County Teaching Hospital, Nyiregyhaza; 8 Balassa Janos Tolna County Hospital, Szekszard; 9 National Institute of Rheumatology and Physiotherapy, Budapest; 10 Dept. of Pediatrics,University of Debrecen; 11 Dept. of Pediatrics, University of Szeged, Hungary

## Background

Several studies on SLE suggest that age at onset modifies the expression of the disease in terms of clinical presentation, pattern of organ involvement and laboratory findings. There are only few data about the age-related differences within the pediatric SLE group.

## Aim

To determine the different clinical manifestations and laboratory characteristics of juvenile SLE (jSLE) in the pre- and postpubertal age in patients fulfilling the 1997 ARA criteria for SLE.

## Methods

Retrospective multicenter analysis of data of 77 jSLE patients, divided into two groups according to the age. Mean age at disease onset was 8.9±1.9 (range 4-11y), follow-up period 7,1±4.3y in the prepubertal (n=30), and 14.7±1.2 (range 11-16y) follow-up period 6.0 ±4.3y in the postpubertal (n=47) group. Various clinical and laboratory parameters were analysed and compared between groups.

## Results

Female:Male ratio was 9:1 in both groups. The most common initial manifestations as butterfly rash occured in 60%/62%, cytopenias 60%/57%, kidney involvement 47%/43%, serositis 27%/23%, in the pre/postpubertal group. Arthritis was significantly more common in the older group (57%/77%). General symptomes like fever 47%/57%, weight loss 23%/30%, increased infection rate 20%/13% were also similar in the two age groups, as did ANA, anti-dsDNA, anti-Sm, anti-SSA, anti-SSB positivity and low C3 and C4 levels. Anti-RNP antibody was more common in the younger ages (33%/10%). Mean SLEDAI score was 12 in both groups. Zero SLICC damage index is significantly more common (68%/43%) in the older group after comparable follow-up period.

## Conclusion

There are no prominent clinical differences except arthritis at onset and higher percentage of 0 SLICC damage score in older jSLE patients as compared to youngers.

